# Supplementary material for: Causal interplay between lactose intolerance and gut microbiota: a combined bidirectional Mendelian randomization and in vivo validation study
Source: Front Nutr. 2026 Jun 1;13:1803337. doi: 10.3389/fnut.2026.1803337 (PMC13265576; doi:10.3389/fnut.2026.1803337)
Supplement: Supplementary file 1 [file Data_Sheet_1.zip › supplementary materials/Forward/leave one out cross validation/ebi-a-GCST90027471.finngen_R12_E4_LACTONAS.pdf]

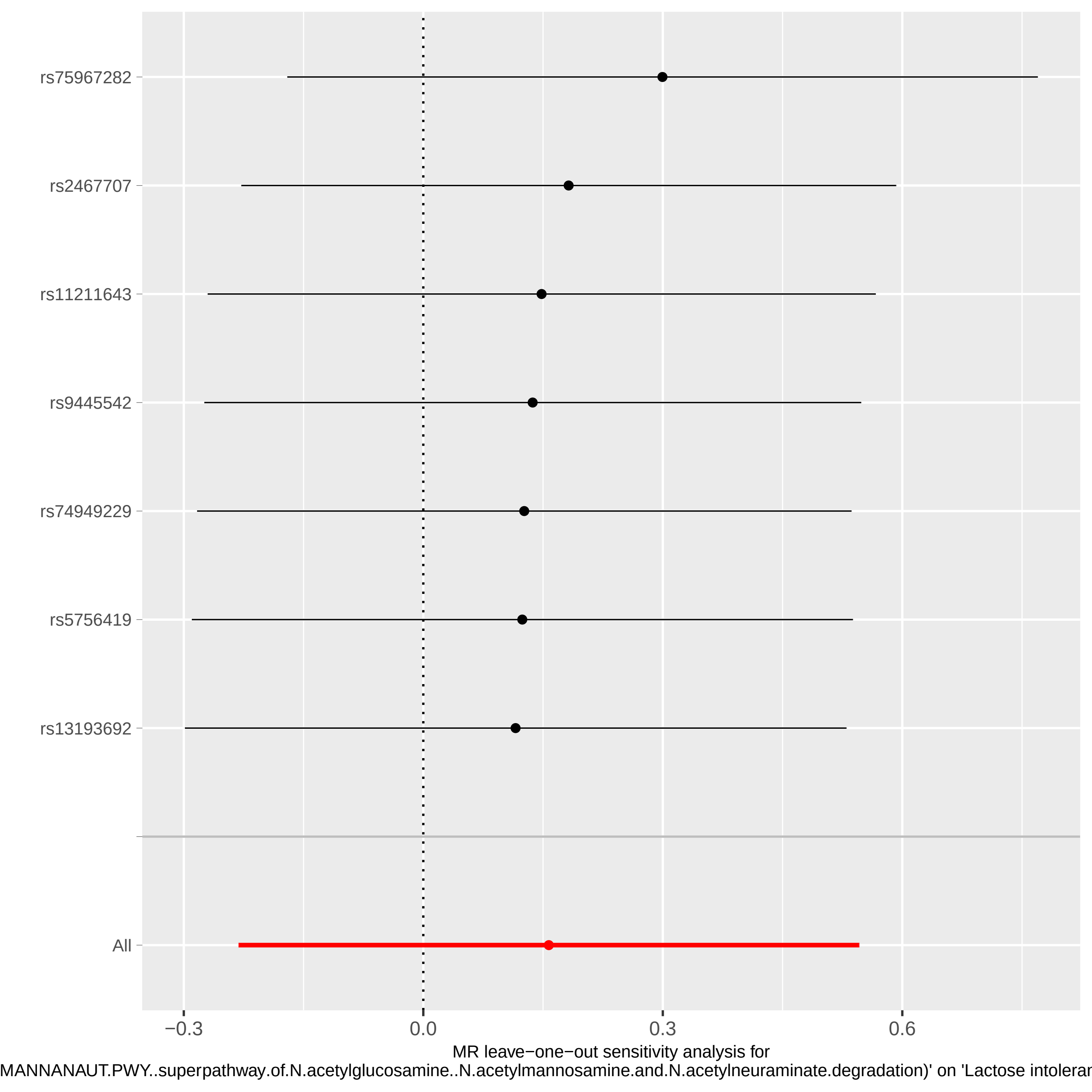
rs75967282
rs2467707
rs11211643
rs9445542
rs74949229
rs5756419
rs13193692
All
−0.3
0.0
0.3
0.6
MR leave−one−out sensitivity analysis for
MANNANAUT.PWY..superpathway.of.N.acetylglucosamine..N.acetylmannosamine.and.N.acetylneuraminate.degradation)' on 'Lactose intoleran
